# Micropreconcentrator in LTCC Technology with Mass Spectrometry for the Detection of Acetone in Healthy and Type-1 Diabetes Mellitus Patient Breath

**DOI:** 10.3390/metabo4040921

**Published:** 2014-10-10

**Authors:** Artur Rydosz

**Affiliations:** Department of Electronics, AGH University of Science and Technology, Av. Mickiewicza 30, Krakow 30-059, Poland; E-Mail: artur.rydosz@agh.edu.pl; Tel.: +48-12-617-30-39; Fax: +48-12-633-23-98

**Keywords:** breath analysis, low temperature cofired ceramics (LTCC) technology, micropreconcentrators, exhaled acetone measurements

## Abstract

Breath analysis has long been recognized as a potentially attractive method for the diagnosis of several diseases. The main advantage over other diagnostic methods such as blood or urine analysis is that breath analysis is fully non-invasive, comfortable for patients and breath samples can be easily obtained. One possible future application of breath analysis may be the diagnosing and monitoring of diabetes. It is, therefore, essential, to firstly determine a relationship between exhaled biomarker concentration and glucose in blood as well as to compare the results with the results obtained from non-diabetic subjects. Concentrations of molecules which are biomarkers of diseases’ states, or early indicators of disease should be well documented, *i.e.*, the variations of abnormal concentrations of breath biomarkers with age, gender and ethnic issues need to be verified. Furthermore, based on performed measurements it is rather obvious that analysis of exhaled acetone as a single biomarker of diabetes is unrealistic. In this paper, the author presents results of his research conducted on samples of breath gas from eleven healthy volunteers (HV) and fourteen type-1 diabetic patients (T1DM) which were collected in 1-l SKC breath bags. The exhaled acetone concentration was measured using mass spectrometry (HPR-20 QIC, Hiden Analytical, Warrington, UK) coupled with a micropreconcentrator in LTCC (Low Temperature Cofired Ceramic). However, as according to recent studies the level of acetone varies to a significant extent for each blood glucose concentration of single individuals, a direct and absolute relationship between blood glucose and acetone has not been proved. Nevertheless, basing on the research results acetone in diabetic breath was found to be higher than 1.11 ppmv, while its average concentration in normal breath was lower than 0.83 ppmv.

## 1. Introduction

Breath analysis has been developing for many years and there have been many instances of research into its potential for diagnosing diseases, e.g., diabetes [1,2,3,4,5]. In general, diabetes is diagnosed on a basis of glucose concentration in blood. A non-invasive and painless method for diagnostic, preventive and monitoring diabetes is still needed. Breath analysis could be faster, cheaper, more flexible and comfortable than blood analysis. Many researchers around the world have been searching for an one-to-one correspondence between a single exhaled compound and a given plasma metabolite [6,7]. Recently, breath analysis has concentrated on acetone as a biomarker of diabetes [8,9,10]. However, the results obtained have often been inconclusive [11]. An overview of the studies of breath analysis in diabetes, focusing on the breath metabolites and their potential utilization in clinical applications, is widely discussed and presented [12]. Careful studies using an appropriate trace gas analytical methods are necessary to the same extent as certified sampling procedures to reduce different outcomes for nominally similar analytical methodologies. The sampling guidance for the breath research community is one of the challenge of the International Association of Breath Research (IABR).

The exhaled acetone is usually in the range of 0.2–1.8 ppmv for healthy people, and in the range of 1.25–2.4 ppmv for people with diabetes [13]. Based on literature review, exhaled breath acetone measurement in its developmental stage is currently characterized by intensive studies and creation of experimental prototype devices [14,15]. Unfortunately, portable devices for exhaled acetone measurements remain currently unavailable. Acetone and other volatiles in breath are present in nanomolar quantities. In order to measure such low concentrations the laboratory systems are applied, *i.e.*, proton transfer reaction mass spectrometry (PTR-MS) [16], atmospheric pressure chemical ionization mass spectrometry (APCI-MS) [17], gas chromatography-mass spectrometry (GC-MS) [18], selected ion flow tube mass spectrometry (SIFT-MS) [19,20]. All mentioned methods are in fact very expensive and require well-qualified personnel, therefore their use is exclusively restricted to laboratories.

Breath analysis as a supplementary tool for diagnosing and monitoring diabetes makes sense only in case of utilization of portable analyzers. However, commercially available gas sensors are developed for measuring samples at several tens part per million (ppm). One of the cheap and very effective methods to increase the limit of detection, in order to measure such low amounts of volatiles, is the use of a micropreconcentrator structures. During a study on T1DM subjects and healthy volunteers, the author aimed to establish the accuracy, repeatability and selectivity response for measurement of exhaled acetone using the micropreconcentrator in low temperature cofired ceramics (LTCC) technology with HPR-20 QIC MS. Based on a literature review, the proposed micropreconcentrator structure in LTCC technology is a novel solution and it have not yet been investigated. It could be an alternative to the commonly presented and discussed preconcentrators (micropreconcentrators) in silicon and silicon-glass technology. In the present study, the exhaled acetone concentrations were measured with micropreconcentrator coupled with mass spectrometry (MS) as a very sensitive detector. Furthermore, the proposed micropreconcentrator can be used with commercially available gas sensors.

## 2. Experimental Section

All experiments involving human subjects were performed according to the “Declaration of Helsinki” and in accordance with Polish law. All patients and volunteers declared a written consent to participate in the investigation.

### 2.1. Reference Gas and Breath Samples

As a reference gas the certified acetone in the concentrations of 80 ppmv, 8 ppmv and 0.8 ppmv (Air Products, Warszawa, Poland) was used. As the exhaled human breath (37 °C) is almost saturated with water, the reference acetone samples were humidified using the Drechsel’s bottle filled with water. The humidity level was controlled by homemade software (LabView), a humidity sensor (SHT25, Sensirion, Stäfa, Switzerland) and mass flow controllers (MKS Instrument, Munchen, Germany). The breath samples were collected with a FlexFoil Plus SKC^®^ (Chicago, IL, USA) Breath-gas Analysis Bag of 1.0 L volume. The bags were stored at room temperature (25 °C), filled with a single exhalation and warmed up to 45 °C for 3 min in order to avoid condensation. Prior to use, the new bags were cleaned three times by using pure (99.9999%) nitrogen. Additionally, to remove any contaminations after each measurement the bags were purified with pure N_2_.

#### 2.1.1. Breath Measurements with Volunteers

Eleven healthy volunteers with no history of any respiratory disease participated in the research, including one (male) heavy smoker, and one pregnant woman. There were five women with an average age of 31 years old and six men with average of 33 years old. Volunteers attended ten visits within two weeks, all visits occurred in the Department of Electronics AGH, Krakow, Poland. The analysis of exhaled acetone was performed between 8:00 am and 3:00 pm for nine volunteers, and between 3:00 pm and 8:00 pm for another two. Subjects of the experiment refrained from eating or drinking for at least two hours before being tested. After five minutes of rest, the subjects were asked to inhale moderately and then to exhale in the 1-l breath bags and again after 2 h following the consumption of 75 g of glucose. A sample of ambient air was collected at the same time. After collection, gas samples in the breath bags were analyzed using the MS with micropreconcentrator structure. The glucose in the blood was measured using a commercial glucometer Acuu Check Active (Roche Diagnostics^®^, Basel, Switzerland). The blood glucose measurement as well as breath acetone measurement were performed at least two times to avoid any incidental results.

#### 2.1.2. Breath Measurements with Diabetes Patients

Fourteen patients diagnosed with type-1 diabetes were asked to breathe into breath bags and measured glucose in blood using their own glucometers. There were eight women with an average age of 31 years and six men with an average age of 25 years. Ten patients used Acuu Check Active (Roche Diagnostics^®^, Basel, Switzerland), four patients used One Touch Mini Select (LifeScan, Inc.^©^, Milpitas, CA, USA), Contour TS (Bayer GmbH^®^, Leverkusen, Germany), Acuu Check Go and Acuu Check Performa (Roche Diagnostics^®^, Basel, Switzerland), respectively. The breath sample procedure was the same as for the healthy volunteers.

### 2.2. Apparatus

**Figure 1:**
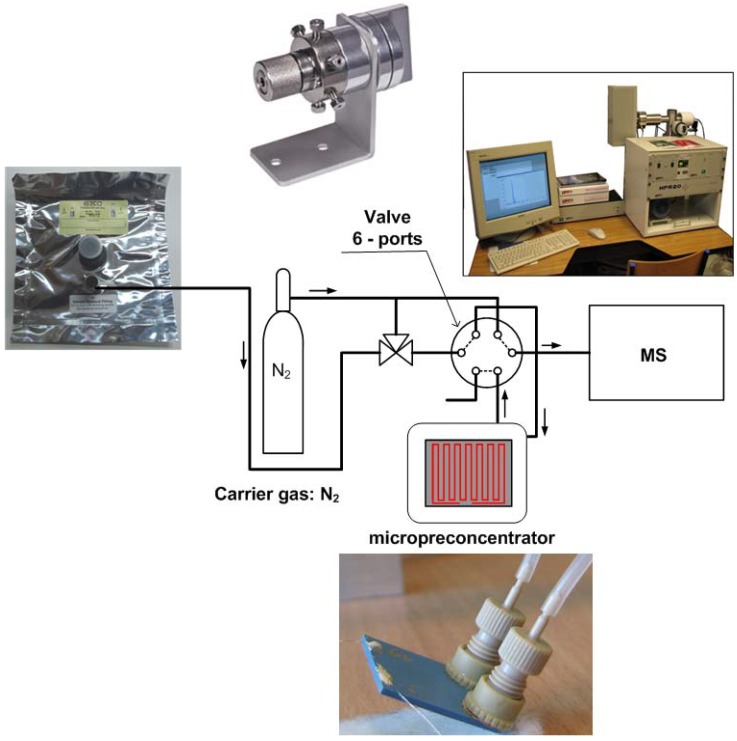
Schematic view of the measurement system based on micropreconcentrator in low temperature cofired ceramics (LTCC) technology and HPR-20 QIC mass spectrometry for exhaled acetone analysis.

**Figure 2:**
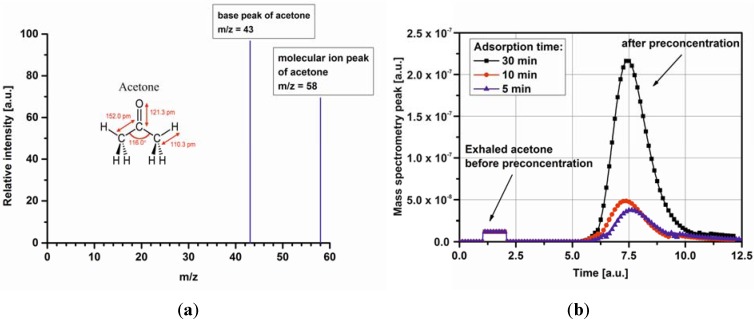
MS spectrum of acetone (**a**), a result of multiple ion detection (MID) analysis of mass spectrograph for the exhaled acetone and acetone after preconcentration for three different adsorption times: 5 min, 10 min, 30 min (**b**).

The HPR-20 QIC real time gas analyzer (Hiden Analytical, Warrington UK) with a 6-ports Valco Injector (VICI Instruments Co. Inc., Schenkon, Switzerland) was used for all the measurements. The micropreconcentrator design and fabrication process was precisely reported in [21]. A schematic view of the measurement setup is presented in Figure 1. The instrument was calibrated using a calibration gas mixture as well as the certified acetone concentrations. As the carrier gas, pure N_2_ was employed and the injector was maintained at 180 °C. MS interface temperature and the ionization voltage were set at 250 °C and 70 eV, respectively. The acquisition range in SEM detector was set from 10^−7^ Tr to 10^−13^ Tr. The mass spectrometry was operated with a multiple ion detection (MID) mode set at *m/z* of 43 (acetone) and 58 (acetone), see Figure 2a. A scan rate of 1 scan/s was applied. The data collection was made with MASsoft Professional Software (Hiden Analytical, Warrington, UK).

### 2.3. Experimental Methods and Conditions

The experimental procedure consisted of few stages. Firstly, the micropreconcentrator was filled with Carboxen-1018. It is a hydrophobic adsorbent material with a large surface area and grain diameters suitable to channel dimensions. It is also recommended by the company as the best adsorbent material for breath analysis. Before using the micropreconcentrators, the adsorbent material have to be activated. The author used typical time-temperature profiles for adsorbent activation. The activation temperature changed in the range of 100 °C–400 °C and the total activation time equals approximately 3 h. After preparing the micropreconcentrator the measurement setup was calibrated. The preconcentration process is precisely described in [22]. Based on the previously obtained results, gas flow of 25 mL/min was passed through the active material during 5, 10 and 30 min. At the end of each measurement the gas lines were purged with N_2_. Then, an electrical pulse was applied to the heater of the device in order to reach a desorption temperature of 220 °C. Figure 2b shows mass spectrometry peak for the acetone taken from an one T1DM subject before and after preconcentration. In the present study, a concentration factor (CF) is defined as the ratio of gas preconcentration after and before preconcentration process (*i.e.*, the ratio of peak area before and after desorption). The obtained concentration factor is around 3.1, 5.85, 16.35 at 5 min, 10 min, and 30 min adsorption time, respectively.

## 3. Results and Discussion

### 3.1. Detection of Acetone in Breath without Preconcentration

Acetone concentrations in 14 diabetic patients and 11 healthy volunteers were summarized in Table 1. Both groups were examined 10 times. However, Table 1 only shows the results for the lowest and the highest blood glucose concentration obtained during the measurements. The median acetone in the healthy group was 0.63 ± 0.12 ppmv, while in diabetic patients breath was 2.08 ± 0.47 ppmv. The acetone concentration in normal breath was ranged from 0.49 to 0.83 ppmv. The measurement results confirmed the statement that the exhaled acetone concentration for non-diabetic is less than 1 ppmv. Breath acetone concentrations from the diabetes patients were from 1.11 to 3.11 ppmv. The obtained results are in accordance with the results reported in [12,23,24].

**Table 1:** The information of diabetic patients and the breath acetone level.

Subject number	Group	Sex ^a^	Age	Years of diabetes	Breath Acetone (ppmv)	Blood glucose (mg/dl)
1 ^b^	Healthy volunteer	M	43	X	0.49	91
					0.56	111
2 ^c^		F	30		0.57	86
					0.47	132
3		M	28		0.63	101
					0.83	137
4		M	41		0.74	74
					0.81	123
5		F	32		0.65	103
					0.73	150
6		M	28		0.48	86
					0.69	146
7		F	30		0.59	84
					0.71	136
8		M	27		0.63	102
					0.72	145
9		F	39		0.45	86
					0.80	135
10		F	26		0.48	90
					0.79	140
11		M	29		0.45	86
					0.73	151
12	Patients with diabetes type I	F	33	15	1.52	60
					1.70	168
13		M	23	2	1.62	70
					3.12	196
14		M	22	2	1.80	100
					2.75	231
15 ^d^		F	29	15	2.16	64
					1.30	170
16		M	23	13	1.51	174
					3.12	367
17		F	25	4	1.11	65
					1.79	372
18		F	21	5	1.55	82
					3.09	324
19		F	40	10	1.63	70
					2.70	350
20		F	23	3	1.70	75
					1.85	127
21		M	20	1	1.87	181
					3.12	270
22		M	24	8	1.78	63
					1.87	165
23		F	22	2	1.86	90
					2.41	451
24		F	55	15	1.61	70
					2.98	257
25		M	35	21	1.65	62
					3.09	389

^a^ M: male and F: female; ^b^ heavy smoker; ^c^ in the 15th week of pregnancy;^d^ patient with a reverse trend.

**Figure 3:**
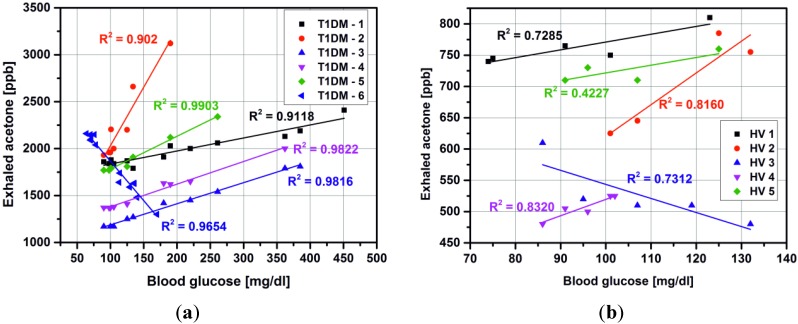
Relations between breath acetone as measured by mass spectrometry and blood glucose for T1DM subjects (**a**) and healthy volunteers (**b**)

**Figure 4:**
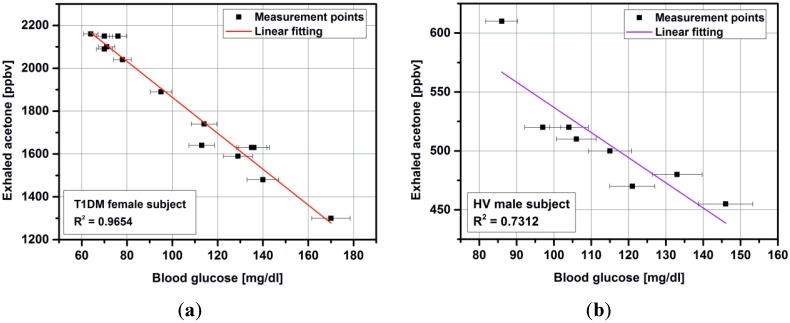
Correlation between breath acetone as measured by mass spectrometry and blood glucose for T1DM subject (**a**) and healthy volunteer (**b**).

Usually, we expect a linear relationship between breath acetone and blood glucose concentration for T1DM (Figure 3a) as well as for healthy volunteers (Figure 3b). However, these studies do not definitively prove a linear and reliable correlation. Therefore, it is clear that as the levels of acetone vary so widely for each blood glucose concentration in each individual, a direct and absolute relationship between blood glucose and acetone does not exist [11]. Furthermore, a reverse trend was observed for one female patient (15 years with diagnosed T1DM) and one healthy volunteer. In both cases, the measurement were performed at the same time of day: 5:00 pm–5:30 pm, the subjects had the same individual diet and resisted of taken any drugs, except insulin by T1DM (NovoRapid, Warsaw, Poland). The correlation coefficient in linear regression was 0.9654 and 0.7312 for T1DM subject and HV, respectively. The obtained results with linear fitting are presented in Figure 4.

### 3.2. Impact of Micropreconcentrator on Breath Acetone Detection

**Figure 5:**
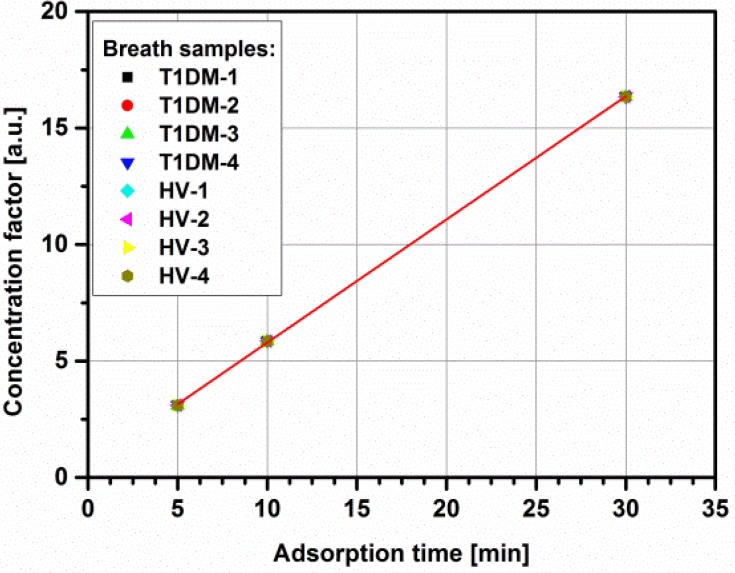
Concentration factor with respect to adsorption time for breath samples taken from T1DM and healthy subjects.

Based on the successful acetone preconcentration with the micropreconcentrator structure in LTCC technology [21] as well as in MEMS technology [22], breath samples taken from T1DM patients and healthy volunteers were determined. As was already stated, breath analysis only makes sense in the case of utilization of portable analyzers. Semiconductor gas sensors are a good alternative to laboratory systems with their low cost, ease of control and compatibility with microelectronic technology, including LTCC technology. The main drawbacks are low selectivity, sensitivity at ppm level and response drift. However, in recent years, research in semiconductor sensors has focused on nanomaterials, which resulted in improved parameters such as: sensitivity, selectivity and stability. The exhaled acetone concentration was in 0.52–3.12 ppmv range in both investigated groups. Unfortunately, it is below a limit of detection (LOD) for commercially available semiconductor acetone sensors. One of the cheapest and very effective method to increase LOD to measure such low amounts of acetone is using the micropreconcentrator structures. It accumulates and concentrates the acetone over an adsorption time period and desorbs mainly based on thermal desorption. Such solutions are common in breath analysis [25,26,27,28]. To start with, the exhaled acetone concentration was measured directly from the breath bags. Then, the preconcentration process started with various adsorption times, *i.e.*, 5 min, 10 min, and 30 min. During the preconcentration process, the breath sample was taken from breath bag with flow rate set to 25 mL/min. The acetone concentration measured after preconcentration was in the range of 1.61–51.01 ppmv. The concentration of the initial gas was enhanced to the ppm range, placing it within the sensitivity range of the available gas sensors. Figure 5 shows the concentration factor with respect to adsorption time for acetone obtained from breath samples: four healthy volunteers and four T1DM subjects. It is obvious that the best results are obtained with a longer preconcentration time. It was previously proved that after 90 min adsorption time, the concentration factor reaches the constant value. However, after 30 minutes adsorption, the acetone concentration measured from diabetic patients breath samples increases approximately 17 times and reaches the LOD for commercially available acetone sensors [29].

## 4. Conclusions and Perspectives

The aim of the presented investigation was the detection of exhaled acetone in healthy and type-1 diabetic patients. The exhaled acetone concentration was in 0.52–3.12 ppmv range in both investigated groups. The breath samples were further determined using micropreconcentrator structure filled with Carboxen-1018 adsorbent material. The adsorption time was reduced to 5, 10 and 30 min. The effect of the concentration factor changes for acetone samples at different initial concentration as well as various flow rates, desorption temperature, *etc.* was investigated in previous work [21]. Therefore, it was essential to investigate how the concentration factor changes for acetone from diabetic and non-diabetic breath (Figure 5). The concentration of the initial gas was enhanced to the ppm range, placing it within the sensitivity range of the available gas sensors. The exhaled acetone higher than 1.11 ppmv was found in diabetic breath and less than 0.83 ppmv in healthy controls. The obtained results proved that type-1 diabetic patients have higher exhaled acetone concentration than healthy people. However, the real potential of breath acetone will rely on its ability to monitor the small changes in breath acetone that parallel changes in blood glucose. It seems that such correlation never exist.
